# Toward accurate single image sand dust removal by utilizing uncertainty-aware neural network

**DOI:** 10.3389/fnbot.2025.1575995

**Published:** 2025-09-10

**Authors:** Bingcai Wei, Hui Liu, Chuang Qian, Haoliang Shen, Yibiao Chen, Yixin Wang

**Affiliations:** ^1^School of Computer Science, Wuhan University, Wuhan, China; ^2^Intelligent Transport Systems Research Center, Wuhan University of Technology, Wuhan, China; ^3^GNSS Research Center, Wuhan University, Wuhan, China

**Keywords:** uncertainty-aware, image restoration, sand removal, hierarchical interaction, feature selection

## Abstract

Although deep learning methods have made significant strides in single image sand dust removal, the heterogeneous uncertainty induced by dusty environments poses a considerable challenge. In response, our research presents a novel framework known as the Hierarchical Interactive Uncertainty-aware Network (HIUNet). HIUNet leverages Bayesian neural networks for the extraction of robust shallow features, bolstered by pre-trained encoders for feature extraction and the agility of lightweight decoders for preliminary image reconstitution. Subsequently, a feature frequency selection mechanism is activated to enhance overall performance by strategically identifying and retaining valuable features while effectively suppressing redundant and irrelevant ones. Following this, a feature enhancement module is applied to the preliminary restoration. This intricate fusion culminates in the production of a restored image of superior quality. Our extensive experiments, using our proposed Sand11K dataset that exhibits various levels of degradation from dust and sand, confirm the effectiveness and soundness of our proposed method. By modeling uncertainty via Bayesian neural networks to extract robust shallow features and selecting valuable features through frequency selection, HIUNet can reconstruct high-quality clean images. For future work, we plan to extend our uncertainty-aware framework to handle extreme sand scenarios.

## 1 Introduction

Adverse weather conditions, including rain, haze, dust, and sandstorms (Shi et al., 2023; Narasimhan and Nayar, 2002; Li et al., 2017b; Shi et al., 2019; Zheng et al., 2021; Zamir et al., 2021, 2022; Wu et al., 2021; Li et al., 2022), present substantial challenges for visual perception systems. Sand and dust are particularly problematic: they obscure critical details like object edges and textures, and directly impair downstream tasks such as object detection and autonomous navigation. This drives significant advancements in image restoration research (Zamir et al., 2021). While deep learning methods, particularly those based on encoder-decoder (Chen et al., 2022) and UNet architectures (Zamir et al., 2022), have demonstrated remarkable progress, they face inherent limitations when handling the complex uncertainty patterns characteristic of sand and dust degradation. Traditional encoder-decoder frameworks (Zheng et al., 2021; Qin et al., 2020; Wu et al., 2021; Zamir et al., 2022), despite their success in general image restoration tasks, exhibit critical shortcomings in extracting comprehensive feature representations from severely degraded images (Zamir et al., 2021), primarily due to their single-level feature extraction paradigm and inadequate uncertainty modeling capabilities.

To address these challenges, we propose a novel framework that integrates uncertainty estimation with shallow feature extraction in a unified, uncertainty-aware architecture. Our approach leverages three key components: (1) Bayesian Neural Networks (BNNs) (Kononenko, 1989; Mackay, 1992; Neal, 2012) for uncertainty-aware shallow feature extraction, (2) a pre-trained encoder (Chen et al., 2022) for robust deep feature extraction, and (3) lightweight decoder modules for initial restoration. The framework further incorporates a frequency-aware feature selection network to dynamically identify and enhance informative features. Following initial restoration, we introduce a shallow feature refinement module that progressively enhances feature representations through thinning operations. Finally, a multi-level feature fusion mechanism synthesizes hierarchical information to generate the final restored image. Extensive experiments on diverse degradation scenarios, including sand, sandstorm, and dust conditions, demonstrate the effectiveness and theoretical soundness of our proposed method.

In summary, the primary contributions of this paper can be categorized into these main aspects:

- To the best of our knowledge, we are the first to utilize uncertainty estimation to characterize various degradation phenomena in single image sand removal, employing the Bayesian neural networks as its implementation. We also first to employ a pre-trained encoder to get deep prior from valuable deep features in single image sand dust removal.
- We introduce a novel frequency selection mechanism for facilitating channel dimensional interaction.
- We propose the Hierarchical Interactive Uncertainty-aware Network(HIUNet) for accurate single image sand dust removal.
- Through comprehensive experiments conducted on our Sand11K dataset, we demonstrate that our proposed method outperforms current state-of-the-art techniques.

In the following section, we will provide the related works of single image sand dust removal.

## 2 Related works

### 2.1 Sand images formulation

The extensively applied physical model elucidating the formation of images perturbed by light transmission through haze (Narasimhan and Nayar, 2002; Si et al., 2022) is conventionally delineated as follows:


(1) $$\begin{array}{l} I\left(p\right)=J\left(p\right)t\left(p\right)+A\left(1-t\left(p\right)\right) \end{array}$$

where *I*(*p*) represents the observed hazy image, *t*(*p*) signifies the medium transmission map, *A* denotes the global atmosphere light, and *J*(*p*) corresponds to the haze-free image. Further, *t*(*p*) can be expressed as: *t*(*p*) = *e*^−β*d*(*p*)^, where β represents the scattering coefficient of the atmosphere, and *d*(*p*) signifies the scene depth between the digital camera and the captured object for each pixel *p* in the image.

**Figure 1:**
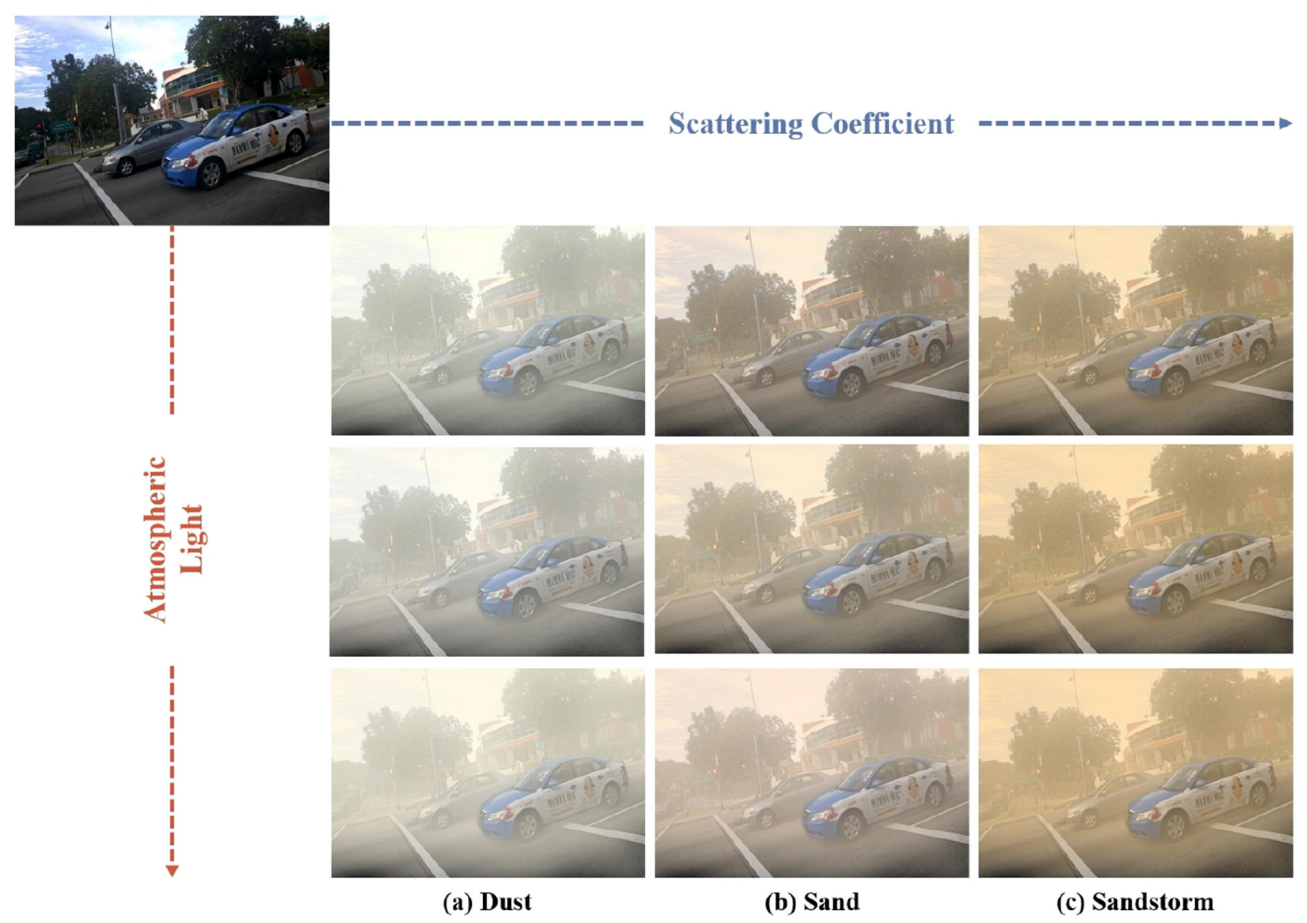
The formulation process of sand and dust images. **(a)** Dust image, **(b)** Sand image, **(c)** Sandstorm image.

In dusty conditions, the different ways that red, green, and blue channels degrade lead to telltale signs like shifts, density changes, and variations over time in the images. It can be formulated as:


(2) $$\begin{array}{l} Â=<A_{R},A_{G},A_{B}> \end{array}$$

where *A*_*G*_ = *k*_1_*A*_*R*_+*b*_1_, *A*_*B*_ = *k*_2_*A*_*R*_+*b*_2_, Â is the global color deviation value of the sand dust image, *b* is the disturbance amount, and *k* is the spatial distribution coefficient of the atmospheric light value of the three basic color spectrums. Based on Equation 2, various sandy images with different degrees of degradation can be synthesized. The detailed formulation process is shown in Figure 1.

### 2.2 Image recovery from degraded weather

Qin et al. (2020) introduce FFA-Net, a feature fusion attention network that significantly advances single image dehazing by effectively integrating channel and pixel attention mechanisms; Zamir et al. (2022) introduce Restormer, an efficient Transformer equipped with a novel design that mitigates the computational complexity of traditional Transformers. Gao et al. (2024) propose a single-stage design, based on a simple UNet architecture, with a mountain-shaped structure. Chen et al. (2022) present NAFNet, an innovative approach that simplifies image restoration by eliminating nonlinear activations, achieving comparable state-of-the-art results with reduced computational complexity.

### 2.3 Uncertainty-aware probabilistic modeling

Data uncertainty in deep learning is a key factor influencing model performance, and probabilistic modeling approaches to address data uncertainty are gaining growing attention. Chang et al. (2020) introduce data uncertainty learning in face recognition, proposing *DUL*_*cls*_ and *DUL*_*rgs*_ methods that simultaneously optimize for feature extraction and uncertainty. Guo et al. (2022) present the Uncertainty-Guided Probabilistic Transformer (UGPT), introducing a novel probabilistic approach to enhance training and inference under uncertainty in action recognition tasks. Wang et al. (2021) introduce a data-uncertainty guided multi-phase learning approach for semi-supervised object detection that effectively leverages unlabeled data across varying difficulty levels. Yang et al. (2021) present UGTR, a pioneering approach that integrates Bayesian learning with Transformer-based Vaswani (2017) reasoning to enhance the model's performance for camouflaged object detection.

### 2.4 Bayesian deep learning

In a conventional neural network, the weights are fixed, which leads to its inability to handle the uncertainty in training data effectively. Therefore, regular neural networks cannot handle the uncertainty in training data well. Unlike the weights in traditional neural network models, the weight parameters in Bayesian neural network (Kononenko, 1989) models are no longer a single deterministic value but rather a probability distribution. The learning process of a neural network can be regarded as a Maximum Likelihood Estimation (MLE) (Mackay, 1992):


(3) $$\begin{array}{l} w^{MLE}=\arg\underset{w}{\max}\log P\left(D|w\right)\\ \;=\arg\underset{w}{\max}\underset{i}{\sum }\log P\left(y_{i}|x_{i},w\right) \end{array}$$

where *D* corresponds to the data used for training, *x*_*i*_ represents the input of each node, *w* represents the weights of each node, and *y*_*i*_ represents the output. And if a prior is introduced for *w*, it becomes a Maximum a Posterior(MAP) (Neal, 2012):


(4) $$\begin{array}{l} w^{MAP}=\arg\underset{w}{\max}\log P\left(w|D\right)\\ \;=\arg\underset{w}{\max}\log P\left(D|w\right)+\log P\left(w\right) \end{array}$$

Unlike MAP estimation which only provides the optimal solution through argmax computation, Bayesian estimation derives the complete posterior distribution *P*(*w*|*D*) for parameters *w*. In Bayesian neural networks, weights are characterized as probability distributions rather than deterministic values. This probabilistic formulation enables explicit modeling of prediction uncertainty, which is particularly beneficial for handling diverse degradation patterns in image restoration tasks by quantifying and propagating data uncertainty through the network.

## 3 Method

### 3.1 Overall pipeline

**Figure 2:**
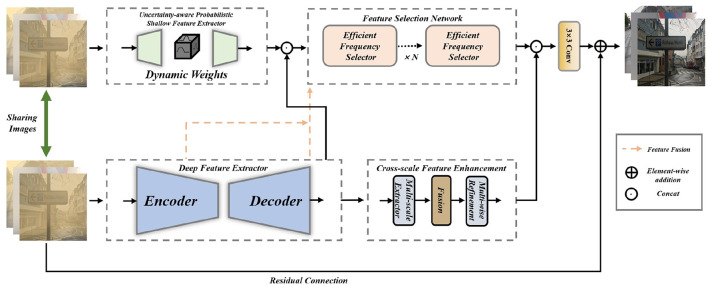
Overall structure of the proposed method. Sharing images are fed into two branches. One branch goes through the shallow feature extractor to generate dynamic weights and then enters the feature selection network with multiple efficient feature selectors. The other branch passes through the deep feature extractor which consists of an encoder and a decoder. After that, the cross-scale feature enhancement module processes these features. The output of the deep feature extractor is also input into the feature selective network. Moreover, the intermediate features of the deep feature extractor are fused into the feature selection network through feature fusion. Finally, the outputs of the two branches are concatenated and then integrated by a convolution layer. Finally, a residual connection is established with the input degraded image to refine the feature integration, culminating in the generation of the final output.

We propose HIUNet, a novel framework for removing dust, sand and sandstorm artifacts from single images (Figure 2). Our method contains five key components: First, to model uncertainty probabilistically, we integrate Bayesian neural networks (Wang and Yeung, 2020; Korb and Nicholson, 2010) that quantify degradation-type uncertainties in the input data. Second, an encoder-decoder architecture extracts deep features, where the encoder utilizes pre-trained weights to capitalize on prior visual knowledge. Third, a Feature Selection Network (FSN) dynamically integrates features from dual extractors through value-based selection, emphasizing informative channels while suppressing redundant ones. Fourth, a Cross-scale Feature Enhancement Module (CFEM) progressively refines the coarse outputs from the deep feature extractor through multi-scale contextual fusion. Finally, the enhanced features from CFEM are concatenated with FSN-selected features to produce the final reconstruction. The comprehensive architecture is visualized in Figure 2. Furthermore, the pseudo-code of our HIUNet can be seen in Algorithm 1.

**Algorithm 1:** HIUNet running process.

Input: Input dusty images *I*_dusty_ (including shared images for input)
Output: Restored clean images *I*_restored_
1: shallow_features←UncertaintyShallowFeatureExtract(*I*_dusty_) ⊳ Uncertainty-aware shallow feature extraction
2: deep_features←DeepFeatureExtract(*I*_dusty_) ⊳ Deep feature extraction via Encoder-Decoder
3: fused_features←Concat(shallow_features, deep_features) ⊳ Feature fusion (Concat operation)
4: selected_features←EfficientFrequencySelect(fused_features, *N*) ⊳ Feature selection with frequency selector (iterated N times)
5: enhanced_features←CrossScaleEnhance(deep_features) ⊳ Cross-scale feature enhancement
6: temp_output←Concat(selected_features, enhanced_features) ⊳ Feature fusion (Concat operation)
7: temp_output←Conv3x3(temp_output) ⊳ 3x3 convolutional layer
8: residual_connected←ElementWiseAdd(temp_output, *I*_dusty_) ⊳ Residual connection
9: *I*_restored_←residual_connected ⊳ Output restored image

### 3.2 Uncertainty-aware probabilistic shallow feature extractor

**Figure 3:**
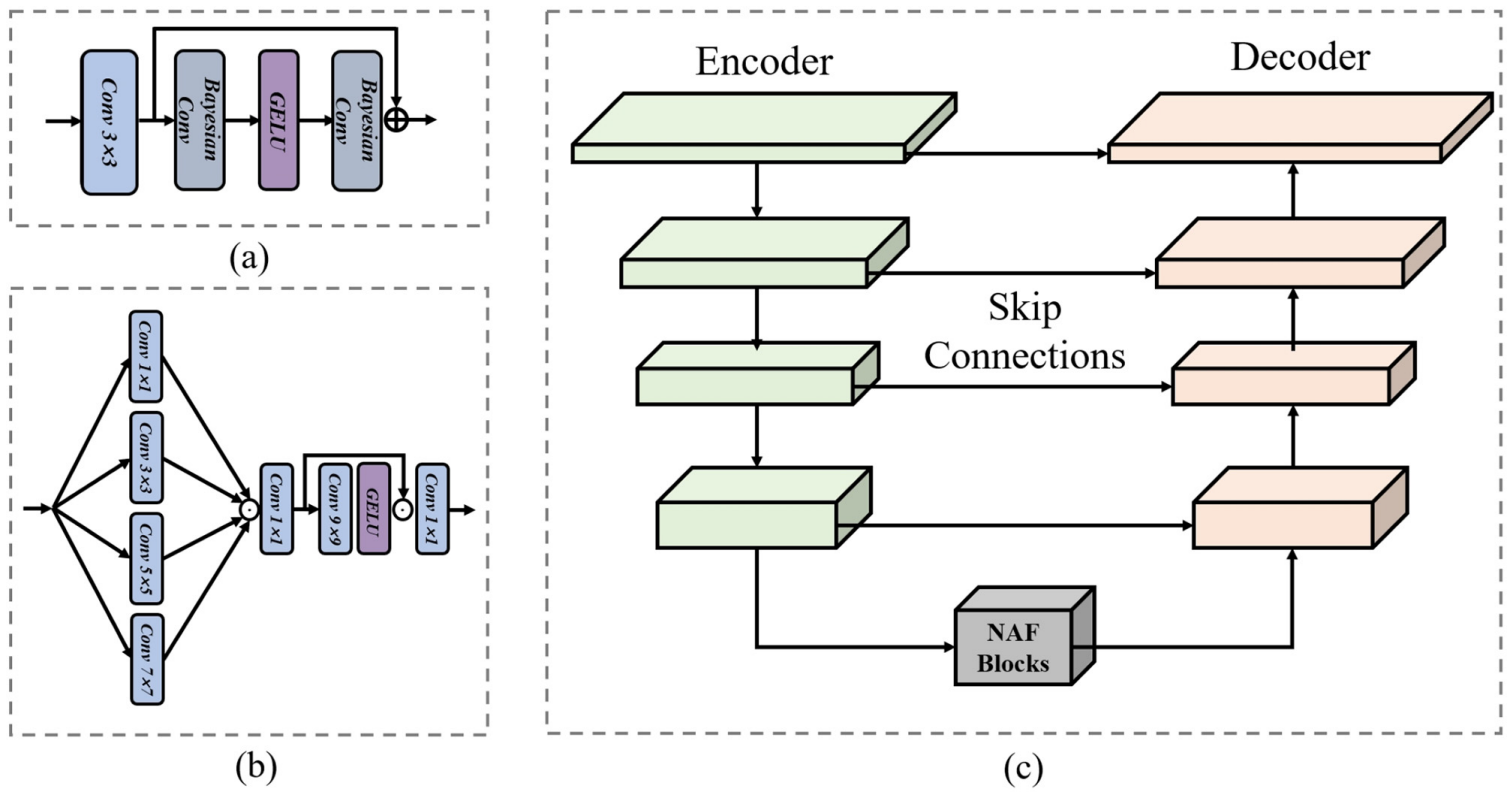
**(a)** Structure of the proposed Uncertainty-aware Probabilistic Shallow Feature Extractor (UPSFE). **(b)** Cross-scale Feature Enhancement Module (CFEM). **(c)** Structure of the employed encoder-decoder.

In our proposed method, Bayesian neural networks (Wang and Yeung, 2020; Korb and Nicholson, 2010) are used as an uncertainty-aware probabilistic shallow feature extractor. Due to the task of simultaneously enhancing the quality of a variety of severe weather-degraded images, the data itself have considerable uncertainty. In order to cope with this situation, we use an uncertainty-aware shallow feature extraction module to solve it. This network is designed to extract features corresponding to multiple types of degradation, providing a more nuanced representation of the input data. For input *x*∈*R*^*h*×*w*×*c*^, the whole process is shown in Equation 5:


(5) $$\begin{array}{l} \hat{x}=B_{2}\left(\theta \left(B_{1}\left(f_{3}\left(x\right)\right)\right)\right)+f_{3}\left(x\right) \end{array}$$

where *x* and $\hat{x}$ are the input and output feature maps, *B*_1_ and *B*_2_ are the BNN layers in our Uncertainty-aware Probabilistic Shallow Feature Extractor (UPSFE), θ is the Gaussian Error Linear Unit. *f*_3_ denotes the convolutional layer with a kernel size 3. The structure of our proposed UPSFE is shown in Figure 3a.

### 3.3 Feature selection network

**Figure 4:**
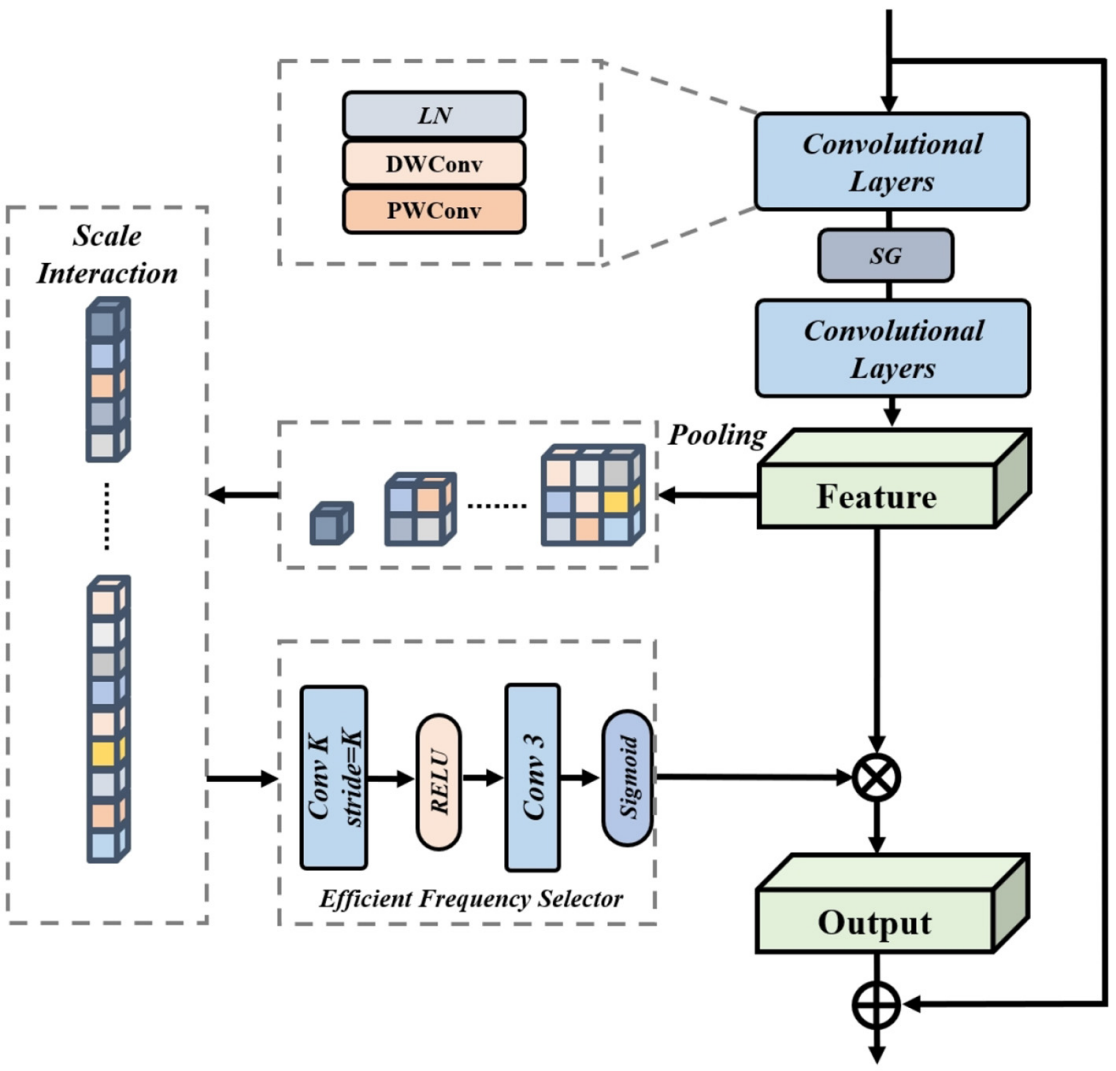
Detailed framework of the feature selection network equipped with our proposed efficient frequency selector.

We introduce the Feature Selection Network(FSN), a module dedicated to the preservation of fine details throughout the image restoration process. Engineered to seamlessly transfer intricate details from the degraded input to the output, FSN enhances the restored image with spatial precision and rich contextual coherence. Furthermore, to sift through complex features and distill those of higher value while suppressing the relatively useless ones, we propose a novel Efficient Frequency Selector (EFS). EFS employs a scale-interacting approach to efficiently integrate features. The following formula mathematically represents the feature selection process:


(6) $$\begin{array}{l} x=DS(SG(DS(x))\\ \hat{x}=\sigma (f_{3}(\psi (f_{k}([x_{1},x_{2},x_{3}]))))*x \end{array}$$

where *DS* and *SG* are depth separable convolution and simple gate, σ and ψ are Sigmoid and ReLU activation functions. The convolutional layer with a kernel size *k* is denoted as*f*_*k*_. [·, ·]denotes the concatenation operation. The detailed framework of our proposed Frequency Selector is shown in Figure 4.

### 3.4 Cross-scale feature enhancement module

In our proposed method, we also utilize the output of deep feature extraction to calculate the loss for the entire network. To refine this output, we introduce a cross-scale feature enhancement module. Firstly, we perform convolution operations on features of multiple scales, enabling them to be concatenated. Next, we apply a dimension reduction operation, followed by integrating the channel dimension characteristics through convolution operations on the large-scale channel dimension. Finally, we stitch together the coarse features after concatenation with the refined channel dimension features and assign elements one by one using pixel-scale convolution. The following formula can represent the entire operation:


(7) $$\begin{array}{l} x=f_{1\times 1}^{c}\left(\left[f_{1\times 1}^{c}\left(x\right),f_{3\times 3}^{c}\left(x\right),f_{5\times 5}^{c}\left(x\right),f_{7\times 7}^{c}\left(x\right)\right]\right)\\ x=f_{1\times 1}^{c}\left(\left[\psi \left(f_{9\times 9}^{c}\left(x\right)\right),x\right]\right) \end{array}$$

where $f_{n\times n}^{c}$ is a convolution layer with *n* kernel and *c* channel, and the structure of this part is shown in Figure 3b.

### 3.5 Pre-trained prior deep feature extractor

In image restoration, the UNet architecture is widely utilized. The encoder serves as a feature extractor, while the decoder is responsible for restoring the image resolution. However, most existing UNet-based methods train the entire Encoder-Decoder together, without separately processing the encoder and decoder. We propose a novel method that leverages a pre-trained encoder for feature extraction for deep prior inspired by previous work (He et al., 2022). The pre-trained encoder is pre-trained on our proposed Sand11K dataset for 200 epoches. We split the UNet into two parts, loading only the encoder's weight. Additionally, we have eliminated some feature processing blocks in the middle of the Encoder-Decoder (Chen et al., 2022), as we believe it to be redundant. Moreover, we have improved the decoder to improve its sensitivity to the texture of features by adding Spatial Attention (Woo et al., 2018) as texture perception mechanisms. The formulation of this whole module is expressed through the following formula:


(8) $$\begin{array}{l} \bar{x}=Dec\left(Mid\left(Enc\left(x\right)\right)\right) \end{array}$$

where *Enc*, *Mid* and *Dec* are Encoder, Middle Blocks and Decoder, respectively, and the structure of Encoder-Decoder employed in this paper is shown in Figure 3c.

### 3.6 Loss function

In order to train all the neural networks involved in this paper in a simple and fair way, we use the Charbonnier penalty function (Charbonnier et al., 1994) to train all the neural networks, including all the comparison experiments and ablation studies. Charbonnier penalty function (Charbonnier et al., 1994) is more tolerant of small errors and holds better convergence during training (Jiang et al., 2020). Charbonnier penalty function (Charbonnier et al., 1994) is shown in:


(9) $$\begin{array}{l} L\left({\text{I}}^{\prime },\hat{\text{I}}\right)=\sqrt{||{\text{I}}^{\prime }-\hat{\text{I}}|{|}^{2}+\epsilon^{2}}, \end{array}$$

where **I**′ is the restored image, $\hat{\text{I}}$ is the ground-truth image, and ϵ = 10^−3^ is a constant in all the experiments.

## 4 Experiments

### 4.1 Implementation details

We implement our framework and other State-Of-The-Art (SOTA) methods using PyTorch with a NVIDIA RTX4090 GPU. We train all the models for 200 epochs with AdamW optimizer. The initial learning rate is set to 1 × 10^−4^, and we employ CosineAnnealingLR to adjust the learning rate. The size of the image patches used for training is 256 × 256. For our proposed Sand11K dataset, following the previous work (Shi et al., 2023), we also synthesize sand and dust images for training, we use 11,985 images as input and 799 ground truth images as labels. The clear images are from PASCAL VOC dataset (Everingham et al., 2015). For testing the performance of learning-based methods, we use 2,397 synthesized sand dust images and real sand dust images.

For the construction process of our Sand11K dataset, we chose the Pascal VOC (Everingham et al., 2015) dataset as clear images. For the training set, we picked 799 sharp images from the Pascal VOC dataset. Then, according to different Atmospheric Light, we synthesized three types of degraded images: dust, sand, and sandstorm, and each type of image contained five degraded images with different degrees according to the Scattering Coefficient. As a result, 1,1985 degraded images were obtained. Then, for the test set, we also selected 799 clear images from the Pascal VOC dataset, and then, according to different Atmospheric Light, we synthesized three types of degraded images: Dust, Sand and Sandstorm. Therefore, the test data totals 2,397 image pairs.

### 4.2 Experimental results

**Figure 5:**
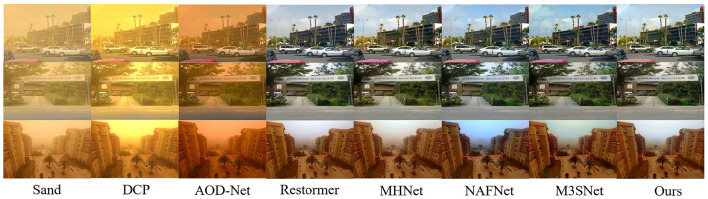
Image restoration results on synthetic and real sand dust images from our proposed Sand11K dataset.

**Figure 6:**
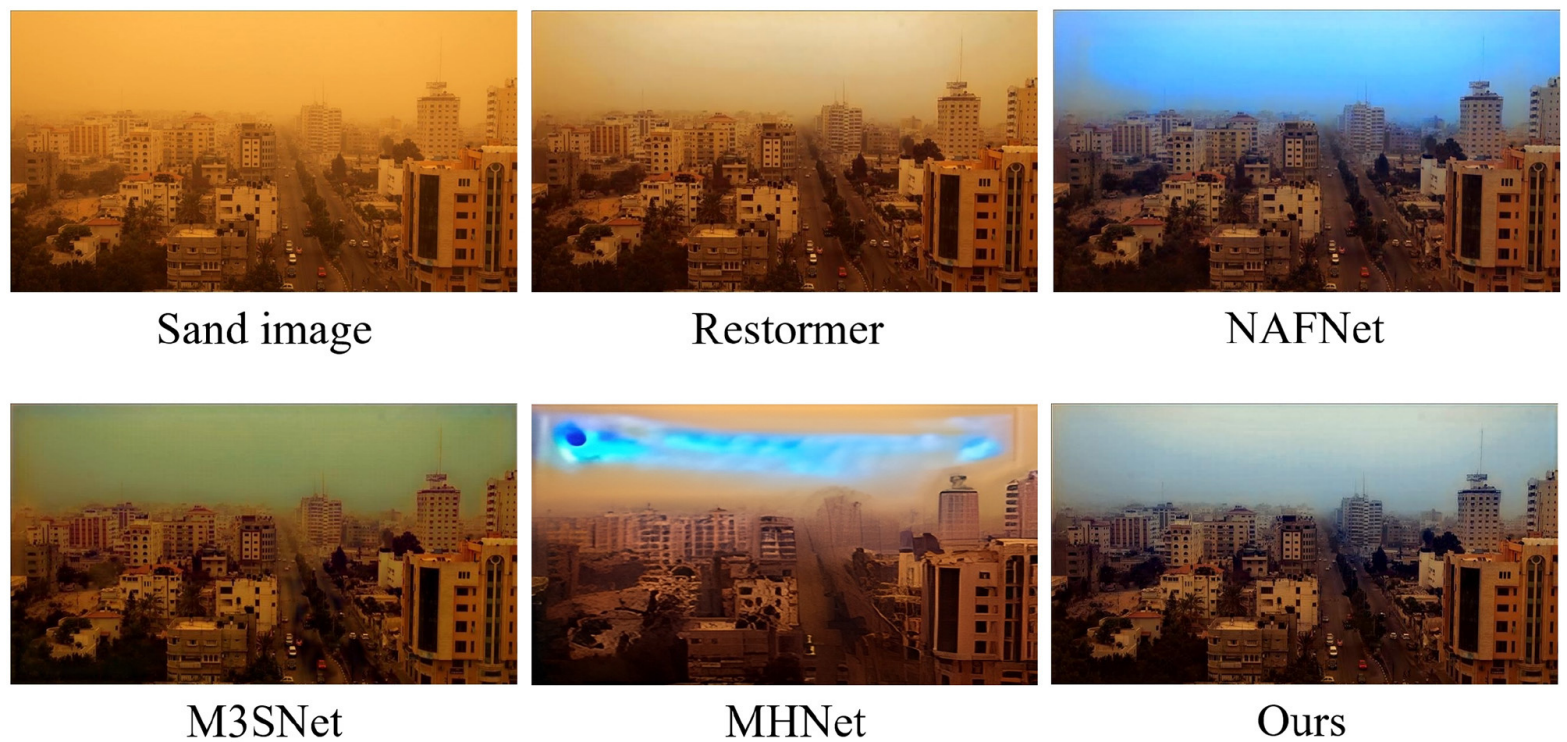
Demonstrating visual effects for scenes with high uncertainty. Methods incapable of learning uncertainty exhibit pattern degradation, whereas our approach showcases a robust capability for uncertainty estimation, resulting in the best visual restoration among the compared techniques.

In the comparative experiments with other state-of-the-art methods, we calculate the scores of Peak Signal-to-Noise Ratio (PSNR), Structural Similarity Index (SSIM), Natural Image Quality Evaluator (NIQE) (Mittal et al., 2012b) and Blind/Referenceless Image Spatial Quality Evaluator (BRISQUE) (Mittal et al., 2012a) using the RGB channelfor the sand dust removal task of a single image. As shown in Table 1, our method achieves significant performance gains that are consistent with or even more than the SOTA methods. For example, our method outperforms the second-best method M3SNet (Gao et al., 2024) 0.27 dB in PSNR for synthetic image sand dust removel. For real-world image sand dust removal, our mehtod ourperforms the second-best method MPRNet 1.81 in BRISQUE. Meanwhile, we provide the computing complexity of all the methods in this paper as shown in Table 2. Furthermore, the visual results shown in Figure 5 are closely aligned with the quantitative findings, substantiating our proposed method's superior image restoration capabilities. As depicted in Figure 6, our method outperforms others in restoring the best visual effects. Unlike alternative approaches that fail to learn uncertainty, leading to pattern degradation such as NAFNet (Chen et al., 2022) and MHNet (Gao and Dang, 2023), our technique successfully reconstructs images with superior visual quality.

**Table 1:** Comparative results on synthetic images and real images of single image sand dust removal.

**Method**	**Synthetic images**		**Real images**	
	**PSNR**↑	**SSIM**↑	**NIQE**↓	**BRISQUE**↓
Sand images	13.60	0.710	3.76	32.22
(TPAMI'10)DCP (He et al., 2010)	-	-	3.55	28.67
(IJISA'26)TTFIO (Al-Ameen, 2016)	-	-	3.46	26.42
(TIP'18)GDCP (Peng et al., 2018)	-	-	2.81	25.15
(arxiv'17)AOD-Net (Li et al., 2017a)	18.88	0.828	4.03	29.51
(CVPR'21)AECRNet (Wu et al., 2021)	19.18	0.820	9.56	24.51
(AAAI'20)FFANet (Qin et al., 2020)	26.53	0.888	3.55	25.15
(CVPR'21)4KDehazing (Zheng et al., 2021)	30.27	0.958	3.89	26.11
(ICASSP'23)Sandformer (Shi et al., 2023)	31.29	0.964	3.24	24.58
(CVPR'21)MPRNet (Zamir et al., 2021)	32.79	0.963	3.55	22.67
(CVPR'22)Restormer (Zamir et al., 2022)	33.22	0.967	3.84	23.63
(arxiv'23)MHNet (Gao and Dang, 2023)	33.28	0.966	3.67	24.33
(ECCV'22)NAFNet (Chen et al., 2022)	33.28	0.966	3.57	22.73
(TVC'24)M3SNet (Gao et al., 2024)	33.31	0.966	3.41	24.95
Ours	33.58	0.968	3.28	20.86

Red/blue text indicates best/second-best comparative results.

**Table 2:** Comparative results on computing complexity of all the methods.

**Method**	**Flops(G)**	**Params(Million)**	**Inference times (ms)**	**Memory cost (Mb)**
(arxiv'17)AOD-Net (Li et al., 2017a)	0.029	0.001	0.134	0.01
(CVPR'21)AECRNet (Wu et al., 2021)	35.64	7.06	3.312	26.94
(AAAI'20)FFANet (Qin et al., 2020)	72.21	4.46	7.228	17.00
(CVPR'21)4KDehazing (Zheng et al., 2021)	103.34	34.55	6.490	131.86
(ICASSP'23)Sandformer (Shi et al., 2023)	2.69	4.28	8.134	16.32
(CVPR'21)MPRNet (Zamir et al., 2021)	37.21	3.64	6.703	13.88
(CVPR'22)Restormer (Zamir et al., 2022)	35.31	26.13	14.796	99.67
(arxiv'23)MHNet (Gao and Dang, 2023)	12.0	17.03	9.683	64.96
(ECCV'22)NAFNet (Chen et al., 2022)	4.07	29.16	6.740	111.24
(TVC'24)M3SNet (Gao et al., 2024)	4.71	16.73	7.59	63.83
Ours	10.17	19.35	11.82	73.97

**Figure 7:**
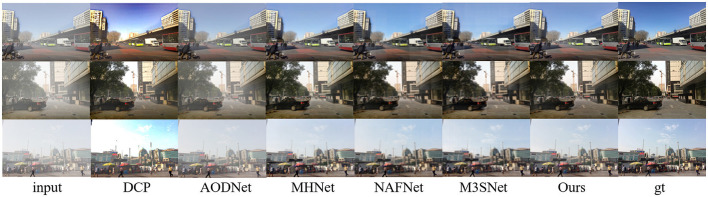
Image restoration results on synthetic and real sand dust images from the public Haze4K (Liu et al., 2021) dataset.

**Figure 8:**
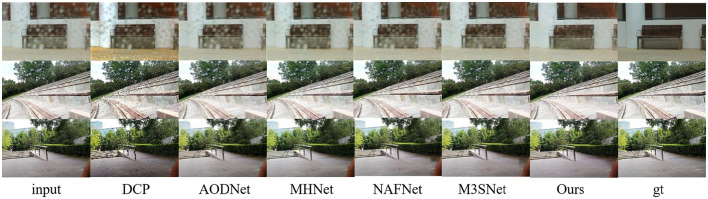
Image restoration results on synthetic and real sand dust images from the public Raindrop-B (Qian et al., 2018) dataset.

In addition, to demonstrate the ability of our proposed method in other similar image restoration tasks, such as image dehazing and image raindrop Removal, we also conducted corresponding experiments using publicly available datasets (Liu et al., 2021; Qian et al., 2018). As shown in Table 3 and Figures 7, 8, our method also performs extremely well in image dehazing and raindrop Removal tasks.

**Table 3:** Comparative results of single image haze removal and rain drop removal.

**Method**	**Haze4K (Liu et al.**, **2021****)**		**Raindrop-b (Qian et al.**, **2018****)**	
	**PSNR**↑	**SSIM**↑	**PSNR**↑	**SSIM**↑
(TPAMI'10)DCP (He et al., 2010)	19.97	0.865	19.20	0.747
(arxiv'17)AOD-Net (Li et al., 2017a)	19.38	0.857	23.64	0.851
(arxiv'23)MHNet (Gao and Dang, 2023)	22.79	0.916	23.74	0.855
(ECCV'22)NAFNet (Chen et al., 2022)	23.11	0.917	24.08	0.860
(TVC'24)M3SNet (Gao et al., 2024)	23.33	0.913	24.15	0.861
Ours	23.56	0.929	24.53	0.871

Red/blue text indicates best/second-best comparative results.

### 4.3 Ablation study

Table 4 presents ablation experimental metrics for UPSFE, Deep Priors, CFEM, and EFS. For the section on ablation experiments, as shown in Table 5, we illustrate the importance of individual components of our method. Tables 4, 5 demonstrate that our proposed component enhances the entire model's performance, underscoring our proposed method's rationality.

**Table 4:** Ablation studies on our proposed UPSFE and EFS.

**Setting**	**wo SFE**	**Conv SFE**	**Linear SFE**	**UPSFE**
PSNR↑	27.17	27.21	27.20	27.30
Setting	CA (Hu et al., 2018)	SCA (Chen et al., 2022)	SRM (Lee et al., 2019)	EFS
PSNR↑	29.27	29.24	29.31	29.37

Red/blue text indicates best/second-best comparative results.

**Table 5:** Ablation experiments of the proposed method.

**UPSFE**	**Deep Prior**	**CFEM**	**EFS**	**PSNR↑**
✓	✗	✗	✗	28.64
✓	✓	✗	✗	29.17
✓	✓	✓	✗	29.28
✓	✓	✓	✓	29.37

Deep Prior means Pre-trained Deep Prior Feature Extractor as described in section 3.5. Red/blue text indicates best/second-best comparative results.

## 5 Conclusion

In this study, we introduce for the first time the concept of uncertainty modeling in the realm of sand dust image recovery. Building upon this, we present the HIUNet framework, which integrates our innovative Efficient Frequency Selector, a Deep Prior Feature Extractor, and a Cross-scale Feature Enhancement Module. Extensive evaluations confirm our method's superiority over state-of-the-art image restoration techniques, with significant advantages in sand-affected scenes. For the future work, we will further advance research on uncertainty estimation modeling and its application in the field of low-level vision.

## Data Availability

The raw data supporting the conclusions of this article will be made available by the authors, without undue reservation.
